# The effects of high-intensity functional training on cardiometabolic risk factors and exercise enjoyment in men and women with metabolic syndrome: study protocol for a randomized, 12-week, dose-response trial

**DOI:** 10.1186/s13063-022-06100-7

**Published:** 2022-03-01

**Authors:** L. E. Smith, G. P. Van Guilder, L. C. Dalleck, N. K. Harris

**Affiliations:** 1 Department of Recreation, Exercise, and Sport Science, Western Colorado University, Gunnison, CO USA; 2 Health and Environmental Sciences Department, Auckland University of Technology, Auckland, New Zealand

**Keywords:** Time-efficient exercise, Minimal dose, Blood lipids, Insulin resistance, Endothelial function, Affective response

## Abstract

**Background:**

Individuals with metabolic syndrome (MetS) are at a greater risk for developing atherosclerotic cardiovascular disease (ASCVD) than those without MetS, due to underlying endothelial dysfunction, dyslipidemia, and insulin resistance. Exercise is an effective primary and secondary prevention strategy for MetS; however, less than 25% of adults meet the minimum stated public recommendations. Barriers often identified are lack of enjoyment and lack of time. High-intensity functional training (HIFT), a time-efficient modality of exercise, has shown some potential to elicit positive affectivity and elicit increased fitness and improved glucose metabolism. However, the effects of HIFT on dyslipidemia and endothelial dysfunction have not been explored nor have the effects been explored in a population with MetS. Additionally, no studies have investigated the minimal dose of HIFT per week to see clinically meaningful changes in cardiometabolic health. The purpose of this study is to (1) determine the dose-response effect of HIFT on blood lipids, insulin resistance, and endothelial function and (2) determine the dose-response effect of HIFT on body composition, fitness, and perceived enjoyment and intention to continue the exercise.

**Methods/design:**

In this randomized, dose-response trial, participants will undergo a 12-week HIFT intervention of either 1 day/week, 2 days/week, or 3 days/week of supervised, progressive exercise. Outcomes assessed at baseline and post-intervention will be multiple cardiometabolic markers, and fitness. Additionally, the participant’s affective response will be measured after the intervention.

**Discussion:**

The findings of this research will provide evidence on the minimal dose of HIFT per week to see clinically meaningful improvements in the risk factors of MetS, as well as whether this modality is likely to mitigate the barriers to exercise. If an effective dose of HIFT per week is determined and if this modality is perceived positively, it may provide exercise specialists and health care providers a tool to prevent and treat MetS.

**Trial registration:**

ClinicalTrials.gov NCT05001126. August 11, 2021.

## Administrative information

Note: The numbers in curly brackets in this protocol refer to SPIRIT checklist item numbers. The order of items has been modified to group similar items (see http://www.equator-network.org/reporting-guidelines/spirit-2013-statement-defining-standard-protocol-items-for-clinical-trials/).

**Table:** 

Title {1}	The effects of high-intensity functional training (HIFT) on cardiometabolic risk factors and exercise enjoyment in men and women with metabolic syndrome (MetS): study protocol for a randomized, 12-week, dose-response trial
Trial Registration {2a and 2b}	ClinicalTrials.gov [NCT05001126]
Protocol Version {3}	Version 2 of December, 16, 2021
Funding {4}	Laboratory equipment and supplies are provided by Western Colorado University’s Exercise and Sport Science Department. Analyses and measurements done at Gunnison Valley Health hospital are donated in-kind by Gunnison Valley Health.
Author details {5a}	Protocol conception and initial design by Smith, Leslie Erin, MS, Western Colorado University^1^, Exercise and Sport Science Department, Gunnison, CO, lesmith@western.edu. Refinement of study protocol and approval of manuscript by Van Guilder, Gary, PhD., Western Colorado University^1^, Exercise and Sport Science Department, Gunnison, CO, gvanguilder@western.edu; Dalleck, Lance, PhD., Western Colorado University^1^, Exercise and Sport Science Department, Gunnison, CO, ldalleck@western.edu; and Harris, Nigel, PhD., Auckland University of Technology^2^, Health and Environmental Sciences Department, Auckland, NZ, nigel.harris@aut.ac.nz.
Name and contact information for trial sponsor {5b}	Investigator initiated clinical trial; LE Smith (principal investigator) lesmith@western.edu
Role of sponsor {5c}	This is an investigator-initiated trial; therefore, the funders played no part in the study design, data collection, study management, data analyses and interpretation, writing of the report, and decision to submit the report for publication.

## Introduction

### Background and rationale {6a}

Atherosclerotic cardiovascular disease (ASCVD) is the leading cause of death worldwide [1–3]. Individuals with metabolic syndrome (MetS) have a twofold higher risk for developing ASCVD [4] and type 2 diabetes [5]. MetS is present in approximately 34% of the US population, now affecting over 100 million individuals [6], nearly doubling in cases since 2014 [7]. A plethora of evidence exists demonstrating that physical activity and exercise reduce risk factors related to MetS and ASCVD [5, 8–10]. Specifically, high-density lipoprotein cholesterol (HDL-C) and triglycerides (TG) respond favorably in a dose-response manner to exercise intensity and duration [11–17]. Additionally, elevated low-density lipoprotein cholesterol (LDL-C) and total cholesterol (TC) are reduced when aerobic exercise reaches intensities between 75 and 90% maximal oxygen consumption (VO_2max_) [11, 14, 15]. Studies that investigated resistance exercise alone found an independent association with favorable changes in several blood lipid markers (TC, LDL-C, HDL-C, TG, TC:HDL-C) [13, 15, 18]. In addition to favorable blood lipid effects, regular resistance training has been found to improve body composition [19], blood glucose levels [20], and blood pressure [21]. Due to the remarkable evidence demonstrating exercise as an ASCVD and MetS risk mitigator, general exercise recommendations have been published internationally [8, 22].

The combined risk factors of high TG and low HDL-C regularly accompany a metabolic alteration in the size and content of the LDL particle [23]. This has been found to be exacerbated in populations with MetS [24] and believed to be more atherogenic than non-altered LDL particles [25, 26]. This alteration manifests as a greater amount of smaller, delipidated LDL particles, which often present as normal or low LDL-C in blood tests [27]. Different blood metrics exist that count the number of these particles (LDL-P or ApoB) and have been found to be better predictors of ASCVD in diseased populations [28–35]. Limited research exists investigating the favorable effects of exercise training on LDL-P and ApoB. Aerobic exercise at intensities of 65–85% VO_2max_ seems to be beneficial in a volume dose-response manner [17], LDL particle size was improved at higher intensities [36], and additional improvements were found when resistance training was added [37].

Endothelial dysfunction, an early event in the development of ASCVD [38, 39], accompanies MetS and its individual risk factors including hypercholesterolemia [40], hypertension [41], and obesity [42, 43]. Improvements in endothelial function by 2–2.8% translates to a reduction in ASCVD risk by 26–36% [44]. Exercise training improves endothelial function even when changes in risk factors are inconsistent [45, 46]. Specifically, aerobic exercise showed improvements in endothelial function in a dose-response manner with intensity and duration [47, 48]. Resistance exercise, on the contrary, demonstrated adverse effects on vascular stiffness, an independent marker of coronary artery disease associated with endothelial dysfunction [49]. In type 2 diabetics, all exercise modalities improved endothelial function, with the low-to-moderate intensity aerobic subgroups improving the most [50].

Despite the plethora of evidence on the benefits of exercise, only 1 in 4 adults in the world meet publicly communicated recommendations [22, 51]. “Lack of time” and “lack of enjoyment” were the reasons found for low exercise participation [52–54]. High-intensity functional training (HIFT) is an abbreviated modality of exercise that has been shown to elicit exercise enjoyment and adherence [55, 56]. HIFT is defined as a training style (or program) that incorporates functional, multimodal resistance movements, performed at relatively high aerobic intensities in interval sets for time, reps, or rounds [57]. HIFT combines aerobic and functional resistance exercise in one time-efficient workout. Furthermore, this modality uses minimal equipment and space, reducing the barrier of having to use a fitness facility [57]. In at-risk populations, HIFT has improved oxygen capacity, insulin resistance, and muscular strength [58], as well as the MetS *z*-score [59]. In healthy populations, HIFT has also improved cardiorespiratory fitness [60, 61], muscular strength and power [62], and waist circumference and agility [61]. Willis et al. found that a 40-min session of HIFT expended an average of 485 kcal [63]. If performed several times per week, HIFT has the potential to meet the weekly recommended energy expenditure to improve health outcomes [19]. Little is known about the effects of HIFT on blood lipid markers, endothelial function, and other risk factors associated with MetS.

The minimum, beneficial dose of HIFT per week has not been investigated. However, minimal doses of the HIFT components: high-intensity aerobic and resistance exercise, have been explored. High-intensity interval training (HIIT) describes unimodal aerobic exercise, performed in brief, intermittent vigorous bursts, interspersed with periods of rest [64]. HIIT resembles the aerobic component of HIFT. When HIIT is performed a minimum of two times per week, this exercise demonstrated benefits in VO_2peak_, waist circumference, thigh cross-sectional area, and quality of life [65]. Additional improvements in body composition and blood lipids were seen only after HIIT was performed three times per week, indicating an influence of frequency [65]. Resistance training, the other component of HIFT, demonstrated superior hypertrophic outcomes after two times per week versus volume-matched exercise one time per week, also indicating an influence of frequency [66, 67]. Research on the dose effects of resistance training on blood lipids, insulin resistance, and endothelial function is minimal; however, performing resistance training less than 1 h per week is associated with a 29% lower risk of developing MetS [68]. With HIFT being a combination of HIIT and resistance training, which both possess certain dose-response characteristics, investigations are needed to determine the minimum weekly amount of HIFT needed to see improvements in endothelial health, blood lipids, and insulin resistance.

Knowing that exercise is a robust tool for mitigating and even eliminating MetS [10], including beneficial effects on lipid profile [11–18] and endothelial function [44–50], but participation in the public recommendations is poor, research on well-accepted modalities, and the minimal dose of such, is needed. If a regular habit of HIFT is achievable, enjoyable, and elicits improvements in health, then HIFT could be a realistic and viable lifestyle intervention for the primary prevention of MetS. Thus, the aim of this study is to investigate the dose-response effects to differing weekly frequencies of a HIFT intervention on blood lipids, endothelial function, insulin resistance, and exercise enjoyment, in a population of sedentary individuals with MetS.

### Objectives {7}

#### Research hypotheses

There is a volume dose-response effect of HIFT on blood lipids, glucose metabolism, endothelial function, fitness, and body composition.

There is an inverse dose-response effect of HIFT on the perception of “exercise enjoyment” and “intention to continue.”

#### Primary research aim

The primary research aim is to explore the dose effects of 3 different weekly volumes of HIFT on ApoB, TG, and cholesterol content of LDL, VLDL, and HDL particles; fasting insulin; fasting glucose; glycosylated hemoglobin (HbA1c); and endothelial function after a 12-week training program.

#### Secondary research aim

The secondary research aim is to explore the dose effects of 3 different weekly volumes of HIFT on cardiorespiratory fitness, body composition, resting blood pressure (BP), and resting heart rate (HR) after a 12-week training program.

#### Tertiary research aim

The tertiary research aim is to investigate the subjective responses of “exercise enjoyment” and “intention to continue” after a 12-week HIFT training program of 3 different weekly volumes.

### Trial design {8}

The present study is a parallel-group, three-arm, exploratory, randomized, dose-response clinical trial. Randomization is at a 1:1:1 allocation ratio for the three arms.

## Methods

### Study setting {9}

The intervention and measurements will take place in Western Colorado University’s (WCU) High Altitude Performance (HAP) Laboratory and Gunnison Valley Health Hospital (GVH) in Gunnison, CO, USA.

### Eligibility criteria {10}

Participants will be included on the condition that they (1) are between the ages of 35 and 65 years old; (2) self-report physical inactivity (less than 30 min per day, 3 times per week, for 3 months); (3) possess at least 3 of the 5 risk factors defining MetS (waist circumference ≥ 102 cm (men), ≥ 88 cm (women), resting BP ≥ 130/85, HDL-C ≤ 40 mg/dl (men) ≤ 50 mg/dl (women), fasting TG ≥ 150 mg/dl, and fasting blood glucose (FBG) ≥ 100 mg/dl); (4) have no diagnosis of or taking medication for heart, lung, kidney, liver, or neurological diseases; and (5) have no medical or orthopedic conditions that prevent them from performing exercises. All exercise and measurements performed in the HAP Lab will be supervised and executed by trained exercise scientists. All measurements performed at GVH will be executed by the technicians at the hospital.

To assess participant eligibility, participants will visit the HAP Lab in a fasted state and complete the initial screening. Participants will be measured for resting BP and HR, resting blood oxygen saturation SaO_2_, height, weight, waist circumference, and abdominal height [8, 69]. Next, 40 μL of capillary blood will be collected via fingerprick and analyzed for TC, LDL-C, HDL-C, TG, and FBG with the Cholestech LDX Analyzer [Abbott Diagnostics, Abbott Park, IL]. TG/HDL ratio will be calculated. Participants will then complete a physical activity and diet questionnaire (Full Block Questionnaire, NutritionQuest, 2014) [70, 71], and all included participants will be asked to maintain their self-reported diet throughout the entire study.

### Who will take informed consent? {26a}

All participants will undergo a process of informed consent and discussion with the principal investigator and complete a health history questionnaire prior to eligibility screening. Written information concerning the needs, benefits, and risks of the trial will be delivered, and a verbal explanation will be given. The discussion will include a check of understanding regarding participation and assurance that the participant accepts or does not accept participation. Participants will also be informed that the allocation of treatment will be random regardless of any personal preference they may have.

### Additional consent provisions for collection and use of participant data and biological specimens {26b}

Biological specimens will not be stored for future use and therefore will not be available for ancillary studies. Future use for all other trial data is included in the original informed consent.

### Intervention

#### Explanation for the choice of comparators {6b}

According to the literature, the investigated dose of HIFT exercise is 3 times per week [55, 58, 60–62]. This dose is in line with the global physical activity guidelines [22]. However, HIIT and resistance exercise, two components of HIFT, have shown health and fitness benefits as little as 2 times per week [64–67]. No studies have investigated the health benefits of HIFT less than 3 times per week. The current study aims to investigate the dose-response effects of differing weekly volumes of HIFT. This choice of comparators seeks to elucidate the minimum amount necessary to elicit clinically meaningful benefits. A non-exercise control group will not be included as to not withhold the benefits of exercise from the study population [72, 73].

#### Intervention description {11a}

For 3 weeks prior to the 12 weeks of training, participants will undergo exercise familiarization 2 days/week. Participants will then be randomized into 1 of 3 exercise dose groups (HIFT1, 1 session per week; HIFT2, 2 sessions per week; HIFT3, 3 sessions per week). Then, 12 weeks of supervised training will commence consisting of three 4-week phases, where intensity, duration of exercise, and recovery time are progressed each phase.

##### Three-week adaptive period

**Fig. 1:**
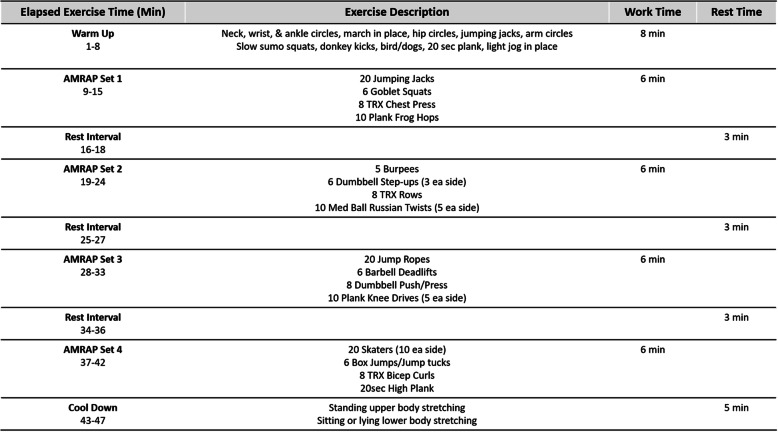
HIFT session example (phase 1). As many rounds as possible (AMRAP)

Twice a week for 3 weeks, participants will visit the HAP Lab to learn proper techniques and familiarize themselves with HIFT exercises. Week 1 will consist of a movement screening to assess the participant’s capabilities of multi-planar, functional movements (squat, hinge, lunge, push, pull, press, and rotation) [74, 75]. Corrections and modifications will be advised to participants, then movements will be trained to ensure proper, safe exercise. If proper movement is not attained within week 1, this familiarization will be extended until satisfaction before progression. Week 2 will consist of learning a structured HIFT routine using minimal load at half of phase 1 volume (see Fig. 1). Week 3 will consist of HIFT rehearsal bouts at ¾ of phase 1 volume (see Fig. 1). Load or resistance will be added to the exercise on an individual basis, aiming for a target perceived effort of ≥ 7 on the Borg Session RPE CR10 Scale (sRPE) [76–79]. Examples of load or resistance are bodyweight, suspension bands, medicine balls, kettlebells, dumbbells, elastic bands, and stability balls. Additionally, participants will be familiarized with the aerobic intensity target (HR ± 5 beats of second ventilatory threshold (VT2)) for each set within the HIFT session. To quantify these intensity targets, HR and sRPE will be measured immediately after each set. Participants will be familiarized with this data collection procedure and intensity target during week 3 [79]. Participants will then be randomized and scheduled for their 12 weeks of training based on their allocated dose (HIFT1, HIFT2, or HIFT3).

##### Twelve-week HIFT training period

Participants will perform their allocated HIFT sessions. All sessions will use asynchronous music playing and be supervised by a Certified Medical Exercise Specialist (CMES) or specifically trained exercise science undergraduate and graduate students when the CMES is unable to attend. These personnel will facilitate exercise instruction, duration and effort monitoring, and data collection and provide standardized encouragement to participants. HIFT sessions will be scheduled on Monday, Wednesday, and Friday in the morning and during the lunch hour, where participants can attend according to their personal schedules and allocated dose group. HIFT session attendance will be organized to ensure between 2 and 5 people at each scheduled time. The training protocol will be divided into three 4-week phases where exercise is progressed in both intensity and volume during each phase, with the last week of each phase being digressed to 60–70% of the volume [80, 81].

Progression of volume will follow the characteristics in Table 1. Progression of load will be individualized for each exercise using the session RPE (sRPE) method, with a target sRPE ≥ 7 (Borg CR10) after each set within the workout [76–79]. The aerobic intensity target (HR ± 5 beats of VT2) will remain throughout. To quantify these intensity targets, HR and sRPE will be measured immediately after each set and recorded by exercise personnel. If a participant’s sRPE falls below 7 or HR falls below the established target, the load will be incrementally increased upon the next training session. Examples of load progression are adding weight or modifying the position of the body to increase resistance or adding elements of velocity to the movement. This personalized method ensures that load/effort is progressed based on each individual’s rate of development and is easily translated into a real-life training session. It also affords the flexibility to be applied to a wide range of exercises and precludes the need for specific maximal strength assessment such as 1RM for subsequent prescription of load percentages.

**Table 1:** Progression characteristics of the 12-week training period

Training parameters	Phase 1		Phase 2		Phase 3	
	Weeks 1–3	Week 4	Weeks 5–7	Week 8	Weeks 9–11	Week 12
Total conditioning duration (min)	36	24	38	26	38	26
Net exercise time (min)	24	16	26	18	28	20
Net recovery time (min)	12	8	12	8	10	6
Work-to-rest ratio	~ 2:1	~ 2:1	~ 2.5:1	~ 2.5:1	~ 3:1	~ 3:1
Work interval (min)	6	4	6.5	4.5	7	5
Rest interval (min)	3	2	3	2	2.5	1.5
Number of exercises	16	16	16	16	16	16
Sets	4	4	4	4	4	4

Each HIFT session will begin with a 10-min warm-up consisting of low-intensity aerobic exercise, dynamic stretching, and movement preparation drills then end with a 5-min cool-down consisting of static stretching. Each HIFT session will consist of 4 sets of 4 functional exercises from the following categories in this order: (1) aerobic, (2) lower body strength, (3) upper body strength, and (4) abdominal/core strength. The exercises will be designed for translatability to various locations using minimal and portable equipment. The amount of load or resistance for each exercise will be determined on an individual basis according to the sRPE target stated above. One round will constitute performing a standardized amount of repetitions or seconds of each functional exercise, back-to-back, in the order listed above. Repetitions will be performed at a standardized tempo (1 s concentric phase/2 s eccentric phase) with proper form. Participants will complete as many rounds as possible (AMRAP) in the prescribed amount of time with recovery in between according to specific work-to-rest ratios. All sessions within a week will be standardized to ensure consistency in dose among the three groups. The total length of each session will be less than 60 min including the warm-up and cool-down. See Fig. 1 for an example of a phase 1 HIFT session. See Table 2 for the list of possible exercises within each category.

**Table 2:** Example of HIFT exercises in each category

Aerobic	Lower body strength	Upper body strength	Abdominal/core strength
Jumping jacks	Goblet squat	TRX chest press	Plank
Skaters	Rear lunge	Push-up	Plank knee drives
High knees jog	Curtsy lunge	TRX row	Side plank
Fast feet with box	Box jumps	Push/press	Russian twist
Kickboxers	Squat jumps	Overhead press	Lateral bends
Butt kick jog	Deadlift	TRX bicep curl	Bicycle crunch
Burpees	Box step-ups	TRX chest fly	Hollow hold
Jump rope	Back/front squat	Triceps dips	Leg lifts
	Wall sit	TRX triceps press	Farmers carry
	TRX pistil squat	Lateral/front raise	TRX plank roll-out
	Jump tuck	Bent-over fly	Exercise ball crunches
		Bent-over row	Plank frog hops
		Negative push-ups	

#### Criteria for discontinuing or modifying allocated intervention {11b}

Participants may choose to stop the HIFT intervention for any reason. There are no special criteria for modifying the allocated intervention. Modification of the exercises within the intervention will be determined on an individual basis if a participant cannot perform the exercise properly. An alternative exercise will be chosen that targets the same muscle group and performed at the same target intensity.

#### Strategies to improve adherence to intervention {11c}

To improve adherence, HIFT exercise will be performed in small groups (2–5 people), which will remain the same throughout the 12-week intervention [82]. Additionally, all HIFT exercises will be facilitated by a CMES or their specifically trained exercise personnel, who will provide accountability, encouragement, and motivation.

#### Relevant concomitant care permitted or prohibited during the trial {11d}

To isolate the effects of the intervention alone, all participants will complete a physical activity and diet questionnaire [70, 71] prior to the start of the study. Participants will be asked to maintain the same behaviors throughout the study, then fill out the questionnaires midway and after the study to verify. Participants will be informed that any additional dietary and exercise interventions will be prohibited throughout the study.

#### Provisions for post-trial care {30}

Understanding that daily physical activity alone can improve health in sedentary populations, upon completion of the study, all participants will be encouraged to continue the regular exercise.

### Outcomes {12}

#### Primary outcomes

##### Blood analysis

Baseline and post-training blood analysis will be measured via venipuncture of the antecubital vein. Five milliliters of blood will be drawn for the lipoprotein metabolism profile (LMPP), 1 mL will be drawn for insulin, 1 mL will be drawn for glucose, 1 mL will be drawn for HbA1c, and 1 mL will be drawn for hematocrit. The LMPP consists of an ApoB count, Lp[a] count, and cholesterol and triglyceride content of each lipoprotein subclass (VLDL, LDL, HDL). Hematocrit is calculated to adjust for plasma volume changes applied to the cholesterol and triglyceride content measures. Glucose and insulin measures will be used to calculate insulin resistance (IR) using the homeostatic model assessment (HOMA) [83, 84]. For each variable, individual change from baseline will be calculated. Data will be aggregated as mean ± standard deviation (SD) for each dose group as well as for male and female subgroups with each dose groups. The mean change ± SD for each variable will be compared between the 3 dose groups and subgroups.

##### Endothelial function

Baseline and post-training endothelial-dependent vasodilation of the non-dominant forearm will be measured using venous occlusion strain gauge plethysmography (EC6 Strain Gauge Plethysmography System & Rapid Cuff Inflation System, Hokanson Inc., Bellevue, WA). Basal endothelial function will be measured for 5 min. During 5 min of reactive hyperemia, peak forearm blood flow (FBF) and 30-s area under the curve blood flow will be quantified. For each variable, individual change from baseline will be calculated and aggregated as mean ± SD for each dose group and subgroups. The mean ± SD for each variable will be compared between the dose groups and subgroups.

#### Secondary outcomes

##### Body composition

Baseline and post-training body composition will be measured via dual X-ray absorptiometry (DEXA). Individual change in total fat mass and lean mass from baseline will be calculated and aggregated as mean ± SD for each dose group and subgroup. The mean ± SD will be compared between the 3 dose groups and subgroups.

##### Self-perceived fitness

Baseline and post-training self-perceived fitness will be measured using the International Fitness Scale (IFIS) [85, 86]. This scale contains five questions with a Likert-type answering option (very poor, poor, average, good, and very good) associated to the elements of physical fitness: cardiorespiratory endurance, muscular strength, speed-agility, and flexibility. Individual change from baseline will be calculated and aggregated as mean ± SD for each dose group and subgroup. The mean ± SD will be compared between the 3 dose groups and subgroups.

##### Graded exercise test

Baseline and post-training cardiorespiratory fitness will be measured via a graded exercise test (GXT) on a power treadmill (CT850, Spirit Fitness, Jonesboro, AR). The GXT will begin with a 5-min warm-up at a self-selected pace, gradually reaching the pace they will maintain throughout the test. A modified Balke and Ware protocol will be used where participants will maintain their constant speed, and incline will be increased by 1% each minute until volitional exhaustion [87]. Maximal HR, VO_2_, and workload will be recorded via HR monitor (Polar F1, Polar USA, Warminster, PA) and metabolic cart (OxyCon Mobile, CareFusion Respiratory Care, Yorba Linda, CA). Participants will rest passively for 20 min after the completion of the GXT, then perform a verification trial to confirm VO_2max_ [88–90]. For the verification trial, participants will walk on the treadmill at a workload of 105% of their maximal workload during the GXT (last fully completed stage) until volitional exhaustion. If the VO_2max_ of the verification bout and GXT are within ± 3%, true VO_2max_ will be considered achieved [88, 90]. If participants are unable to reach VO_2max_, they will be asked to repeat the trial after a 24-h rest. Individual change from baseline will be calculated and aggregated as mean ± SD for each dose group and subgroup. The mean ± SD will be compared between the 3 dose groups and subgroups.

#### Tertiary outcomes

##### Exercise questionnaires

To assess the participant’s level of “enjoyment” and “intention to continue” their allocated HIFT intervention, a questionnaire will be administered post-training. For enjoyment assessment, the Physical Activity Enjoyment Scale (PACES) will be used [91]. The PACES is an 18-item, 7-point, bipolar rating scale. For the intention to continue assessment, two additional items will be added regarding (1) how likely the participant would continue performing that modality (0 = very unlikely, 10 = very likely) and (2) how many days per week the participant would consider performing that modality (0–7 days) [92, 93]. Data will be aggregated as mean ± SD for each dose group and subgroups. The mean ± SD will be compared between the 3 dose groups and subgroups.

#### Participant timeline {13}

Upon recruitment, participants will meet with the primary investigator and provide informed consent and health history. Next participants will be screened for the inclusion criteria at the HAP Lab at WCU. During the screening visit, participants’ height, weight, waist circumference, abdominal height, resting HR, resting BP, and resting SpO_2_ will be measured. Additionally, participants will be measured for a fasted basic blood lipid and glucose panel. Next, participants will complete a physical activity and diet questionnaire (Full Block Questionnaire, NutritionQuest, 2014) [70, 71], and all included participants will be asked to maintain their self-reported diet throughout the entire study.

**Fig. 2:**
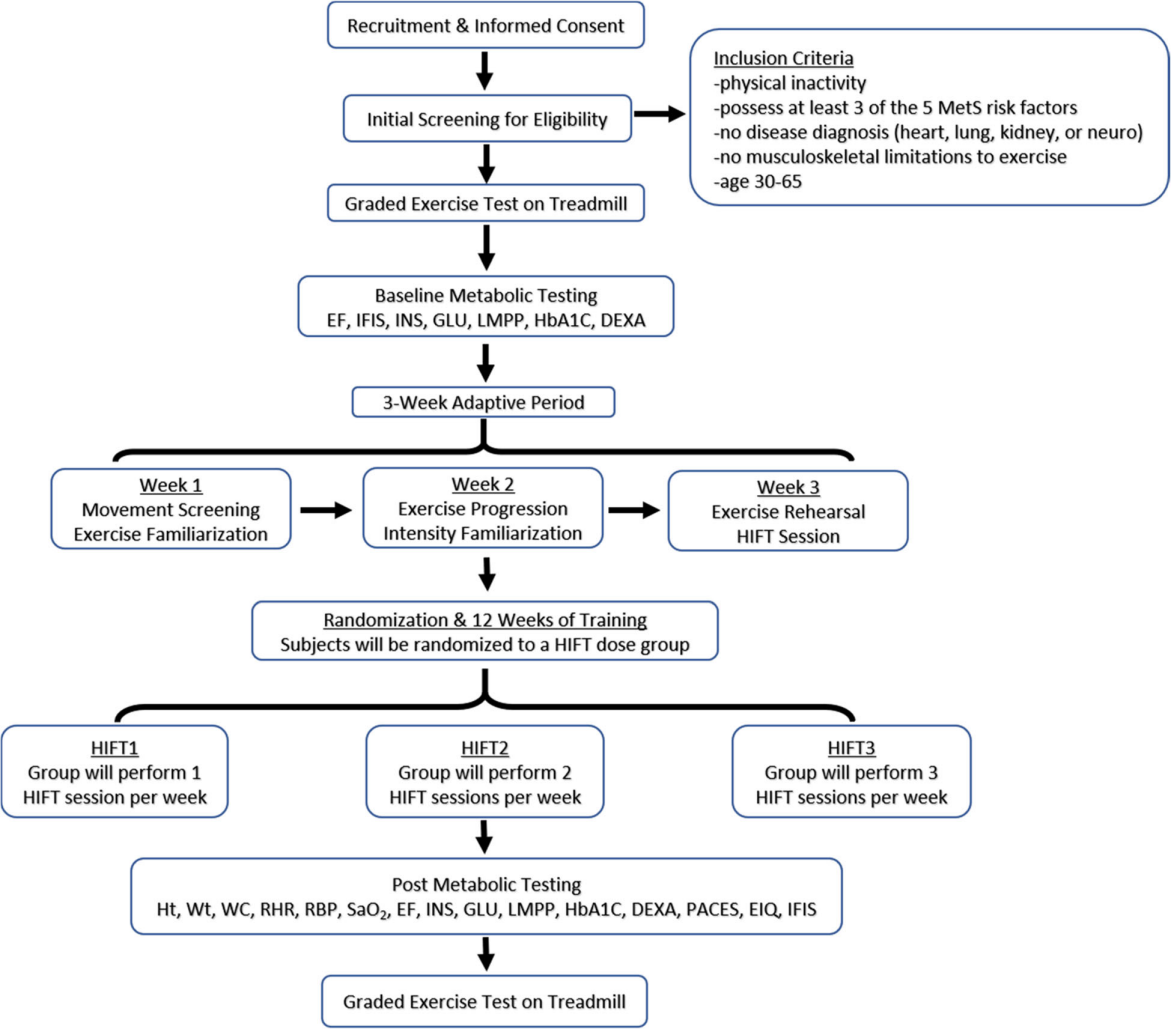
Schematic illustration of the research design. Ht, height; Wt, weight; WC, waist circumference; RHR, resting heart rate; RBP, resting blood pressure; SaO_2_, resting oxygen saturation; EF, endothelial function; INS, blood insulin; GLU, blood glucose; LMPP, lipoprotein metabolism profile; HbA1C, glycosylated hemoglobin; DEXA, dual X-ray absorptiometry; HIFT, high-intensity function training; PACES, exercise enjoyment questionnaire; EIQ, Exercise Intention Questionnaire; IFIS, International Fitness Scale

Participants will begin the study with a baseline GXT immediately after the initial screening. Next, baseline metabolic testing in the HAP Lab and GVH will take place 48–72 h after. This visit will consist of a 12-h overnight fast, baseline endothelial function testing at the HAP Lab, then metabolic marker bloodwork and body composition testing via DEXA at GVH. Additionally, participants will complete the IFIS to assess self-reported fitness [85, 86]. During the following 3 weeks, participants will visit the HAP Lab twice a week for exercise familiarization and adaptation. Participants will then be randomized into 1 of 3 exercise dose groups (HIFT1, 1 session per week; HIFT2, 2 sessions per week; HIFT3, 3 sessions per week) and be scheduled for their exercise sessions for the following 12 weeks. The 12 weeks of supervised training will consist of three 4-week phases, where intensity and duration of exercise are progressed each phase. After the last exercise session, participants will be scheduled for two post-testing visits. The first visit will consist of a 12-h overnight fast, endothelial function testing, PACES and exercise intention questionnaires, and IFIS questionnaire at the HAP Lab, then metabolic marker bloodwork and body composition testing via DEXA at GVH. This visit will be scheduled no sooner than 48–72 h after the final exercise session to ensure acute lipid or glucose responses are not influenced [14, 94–96]. The second visit, 24–48 h later, will consist of the repeated GXT on a treadmill from baseline testing. See Fig. 2 for the schematic illustration of research design and Table 3 for the schedule of the study period.

**Table 3:**
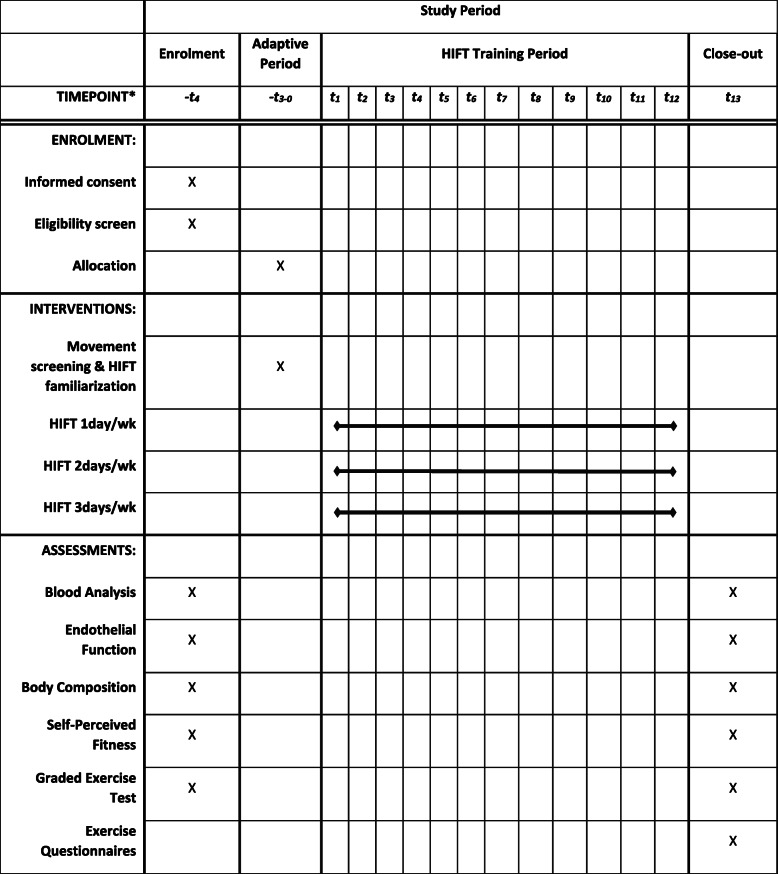
Schedule of the study period

*HIFT* high-intensity functional training

*Each time point represents one week

#### Sample size {14}

The sample size was projected with a change in ApoB as the main outcome variable between HIFT1 and HIFT3. Power calculations (IBM SPSS v25 Armond, NY) assumed a 1:1 equal allocation between HIFT1 and HIFT3. The baseline mean and standard deviations from a previous study [97] and pilot studies for ApoB were ~ 129 ± 25 mg/dL in sedentary adults with elevated cardiometabolic risk. Collectively, using these data, we consider a 15% reduction in ApoB to be clinically meaningful within the HIFT3 group [98]. Assuming a power of 0.90, *α* = 0.05, and an effect size of *d* = 0.80, a paired samples one-tailed *t*-test sample size calculation revealed a sample size of 60 subjects (20 per group) will establish the superiority of HIFT3 versus HIFT1 for pre- to post-differences in the primary outcome ApoB. With an estimated 5% dropout rate, we will recruit 22 subjects/group to obtain the necessary sample size of 60. Therefore, a total of 60 men and women who meet the previously stated inclusion criteria will be recruited and randomized post-baseline assessments into one of the three intervention groups. Data will be collected in cohorts of 12, randomized into intervention groups. This will continue until 20 participants per intervention group have completed the study. This will accommodate dropout within cohorts.

#### Recruitment {15}

Participants will be recruited via advertisement flyers posted in the communities of Gunnison County, social media, word of mouth, employee email lists of WCU and GVH, local newspaper and magazine ads, churches, and through physician referral among the Gunnison County health care providers. To account for the logistical capacity of data collection, participants will be recruited in cohorts of 12 (randomized into intervention groups) where each cohort will complete the entire study before the next cohort begins. This will continue until 20 participants per intervention group have completed data collection.

### Assignment of interventions: allocation

#### Sequence generation {16a}

After completion of baseline testing and adaptive period, participants will be randomly allocated to a HIFT dose group at a 1:1:1 ratio by computer-generated random numbers. This will be conducted by GP Van Guilder, one of the investigators uninvolved with participant recruitment, enrollment, assessments, and exercise training. Randomization will be blocked in cohorts and stratified by sex. The block size will be concealed by this investigator until the primary endpoints have been analyzed.

#### Concealment mechanism {16b}

Throughout the study, randomization will be conducted by GP Van Guilder, utilizing a computer-generated sequence. All allocation sequences will be stored on their personal computer, and sequences will be concealed until the primary endpoints have been analyzed. Treatment allocation will not be released to the participants until after they complete baseline testing and the adaptive period. This investigator will not be involved in recruitment, enrollment, assessments, or any execution of the intervention. Thus, randomization will be conducted without any influence of the principal investigator and research assistants involved directly with recruitment, enrollment, and assessments of participants, as well as delivery of the intervention.

#### Implementation {16c}

GP Van Guilder will communicate the treatment allocation to each participant via a personal email. This communication will only occur after the participant has been enrolled, completed baseline testing, and completed the adaptive period. Thus, allocation communication will be conducted without any influence on recruitment and enrollment.

## Assignment of interventions: blinding

### Who will be blinded {17a}

All data collection sheets and data analysis will be coded by the investigator assigning the interventions in order to blind the primary investigator and research assistants from intervention allocations. Data analysis, data collection, and exercise training will be conducted by the primary investigator and research assistants but not the investigator conducting the allocations. Due to the nature of the intervention, participants will not be blinded to their allocation but are strongly encouraged not to disclose their allocation or the allocation of other participants.

### Procedure for unblinding if needed {17b}

To maintain the quality and legitimacy of the clinical trial, unblinding should only occur in the exceptional circumstance that a participant had an adverse effect from the intervention. In this case, knowledge of the intervention allocation is absolutely essential for further management of the participant’s safety.

## Data collection and management

### Plans for assessment and collection of outcomes {18a}

#### Primary outcomes

##### Blood analysis

Participants will visit GVH for fasted blood draws performed by a trained phlebotomist at baseline and 48–72 h after the exercise intervention. Venipuncture of the anti-cubital vein will be performed, and blood will be collected into heparinized tubes. Five milliliters of blood will be drawn for the lipoprotein metabolism profile (LMPP), 1 mL will be drawn for insulin, 1 mL will be drawn for glucose, 1 mL will be drawn for HbA1c, and 1 mL will be drawn for hematocrit. All blood collected for the LMPP, insulin, and glucose will be frozen at − 80 °C and sent to Mayo Clinic Laboratories in Denver, CO. All results will be communicated back to GVH. The LMPP consists of an ApoB count, Lp[a] count, and cholesterol and triglyceride content of each lipoprotein subclass (VLDL, LDL, HDL). Hematocrit is calculated to adjust for plasma volume changes applied to the cholesterol and triglyceride content measures. Glucose and insulin measures will be used to calculate insulin resistance (IR) using the homeostatic model assessment (HOMA) [83, 84].

##### Endothelial function

Endothelial function will be assessed at baseline and 48–72 h after the last exercise bout of the intervention. Fasted, endothelial-dependent vasodilation of the non-dominant arm will be measured using venous occlusion strain gauge plethysmography (EC6 Strain Gauge Plethysmography System & Rapid Cuff Inflation System, Hokanson Inc., Bellevue, WA). To begin, forearm volume will be measured by the water displacement method. Then, the arm will be elevated to the level of their heart, a pneumatic cuff will be placed around the wrist and upper arm, and a strain gauge will be fixed around their lower arm. Once all measuring devices are attached and ready, the contralateral arm will be used to measure HR and BP until stable resting values are obtained (after 15 min of supine quiet rest). Next, basal blood flow will be measured for 5 min. The wrist cuff will be inflated to 220 mmHg, and the upper arm cuff will cycle between 50 and 0 mmHg every 8 and 7 s for the entire 5 min. Then, the brachial artery will be occluded for 5 min at a pressure of 220 mmHg. At the 4-min mark of the occlusion period, the wrist cuff will be inflated to 220 mmHg again. At the end of the occlusion period, the upper arm cuff will be deflated to assess reactive hyperemia for 5 min. Peak forearm blood flow and 60-s hyperemia blood flow will be quantified.

#### Secondary outcomes

##### Body composition

Participants will visit GVH to be measured for body composition via dual X-ray absorptiometry (DEXA) at baseline and 48–72 h after exercise intervention.

##### Self-perceived fitness

To assess the participants’ self-perceived overall fitness, the International Fitness Scale (IFIS) will be administered at baseline and after the exercise intervention [85, 86]. This scale contains five questions with a Likert-type answering option (very poor, poor, average, good, and very good) associated to the elements of physical fitness: cardiorespiratory endurance, muscular strength, speed-agility, and flexibility.

##### Graded exercise test

Baseline and post-training cardiorespiratory fitness will be measured via a graded exercise test (GXT) on a power treadmill (CT850, Spirit Fitness, Jonesboro, AR) in the HAP Lab. Participants will be instructed on the rate of perceived exertion scale (RPE) and walking protocol, outfitted with a HR monitor (Polar F1, Polar USA, Warminster, PA), connected to a metabolic cart (Oxycon Mobile, CareFusion Respiratory Care, Yorba Linda, CA) and positioned on the treadmill. The GXT will begin with a 5-min warm-up at a self-selected pace, gradually reaching the pace they will maintain throughout the test. A modified Balke and Ware protocol will be used where participants will maintain their constant speed and incline will be increased by 1% each minute until volitional exhaustion [87]. Breath-by-breath gas exchange and continuous HR data will be collected and averaged every 15 s. RPE will be measured in the last 10 s of each minute. Participants will continue until volitional exhaustion. The first and second ventilatory thresholds (VT) will be identified and calculated as the point in which the plotted ventilation rate makes an a-linear increase. Maximal HR, VO_2_, and workload will be recorded. Participants will rest passively for 20 min after the completion of the GXT, then perform a verification trial to confirm VO_2max_ [88–90]. For the verification trial, participants will walk on the treadmill at a workload of 105% of their maximal workload during the GXT (last fully completed stage) until volitional exhaustion. If the VO_2max_ of the verification bout and GXT are within ± 3%, true VO_2max_ will be considered achieved [88, 90]. If participants are unable to reach VO_2max_, they will be asked to repeat the trial after a 24-h rest.

#### Tertiary outcomes

##### Exercise questionnaires

To assess the participant’s level of “enjoyment” and “intention to continue” their allocated HIFT intervention, a questionnaire will be administered 48–72 h after the exercise intervention. For enjoyment assessment, the Physical Activity Enjoyment Scale (PACES) will be used [91]. The PACES is an 18-item, 7-point, bipolar rating scale. For the intention to continue assessment, two additional items will be added regarding (1) how likely the participant would continue performing that modality (0 = very unlikely, 10 = very likely) and (2) how many days per week the participant would consider performing that modality (0–7 days) [92, 93].

### Plans to promote participant retention and complete follow-up {18b}

Exercise supervision and encouragement will take place during every session of the intervention to promote participant retention and safety. Participants will be given a copy of all HIFT workouts and personal health data at the end of the trial which may serve as an incentive to continue the habit of regular exercise on their own.

### Data management {19}

To keep participant personal health data private, all paper data with personal health information, including consent forms and health history documents, will be stored in a secured, locked file cabinet in the principal investigator’s office on WCU’s campus. Only the principal investigator and listed protocol contributors will have access to this personal data.

Participants’ names will be coded to protect identity on all assessments, testing data, and exercise logs. All coded paper data will be stored in a locked file cabinet in the WCU HAP Lab office. These data will be entered electronically at the participating site where the data originated from by the principal investigator. Two trained research data entry personnel will separately verify the electronic copy for validity and range checks. Any errors will be reported to the principal investigator and protocol contributors, and corrections will be made. These electronic data will be stored in a secure (password-protected) database in duplicate, on both WCU’s and AUT’s campus networks to prevent unauthorized access. Incremental back-ups will be performed on a daily basis to prevent data loss.

### Confidentiality {27}

Any potential and enrolled participant information will be kept confidential, only identifiable by primary investigators. Codes will be used instead of names on all documents. For all data collected at WCU and GVH, the standard HIPAA regulations and procedures will be followed. Each participant’s information will be combined with other people taking part in the study when results are shared or presented in materials.

### Plans for collection, laboratory evaluation, and storage of biological specimens for genetic or molecular analysis in this trial/future use {33}

All biological specimens will be destroyed after data is recorded. No specimens will be retained for future use.

## Statistical methods

### Statistical methods for primary and secondary outcomes {20a}

Only data from participants who complete ≥ 80% of total exercise intervention and with no noticeable change in eating (statistically different energy consumption and macronutrient distribution) and physical activity habits will be included [97, 99, 100]. All data will be analyzed using the Statistical Package for the Social Sciences (IBM SPSS v25, Armond, NY). Descriptive data if normal will be presented as mean ± SD, or median and interquartile range if not normal; the significance level of all comparative data will be set as *p* ≤ .05, and normality will be assessed using a Kolmogorov-Smirnov test. If normality exists, parametric tests will be used, if normality does not exist, non-parametric tests will be used. Effect sizes (ES) and confidence intervals (CI) will be calculated on the results of all dependent variables. Paired *t*-tests will be used to evaluate the significant differences between pre- and post-measures of both primary and secondary physiological outcomes within each group. A one-way ANOVA will be used to compare the primary and secondary outcomes pre-post mean change between the three groups. When indicated by a significant *F* value, post hoc analysis will be determined using the Bonferroni adjustment for multiple comparisons. The median, mode, and interquartile ranges of the IFIS and PACES data will be calculated for each of the three groups. Should missing data in outcomes occur, a linear-mixed model will be conducted instead of ANOVA as this test accounts for missing data.

### Interim analyses {21b}

Due to the nature and length of the intervention, no interim analyses will be performed.

### Methods for additional analyses {20b}

To explore whether the outcomes vary significantly between men and women, a secondary analysis will be performed for the stratification variable of sex.

### Methods in analysis to handle protocol non-adherence and any statistical methods to handle missing data {20c}

Participant adherence will be determined by completion of ≥ 80% of total exercise intervention and no noticeable change in eating and physical activity habits, assessed by the Block questionnaires. Should non-adherence occur, outcome variables will be tested using two analysis sets: the intention-to-treat set, considering all randomized participant data regardless of intervention adherence, and the per-protocol set, considering only data from participants who complete ≥ 80% of the total exercise intervention. Analyses will be performed to examine the effect of the different strategies on the conclusions. If intention-to-treat analyses are determined appropriate, reasons for withdrawal will be reported and compared qualitatively. If missing outcome variables occur, imputation of missing data will be considered. To account for missing data, a linear mixed-model analysis will be used to determine if there are differences in the primary outcome variable of ApoB.

### Plans to give access to the full protocol, participant-level data, and statistical code {31c}

This document is the full protocol. Anyone interested in other data or documentation should contact the corresponding author.

### Oversight and monitoring

#### Composition of the coordinating center and trial steering committee {5d}

The trial will be overseen by a trial steering committee (TSM) consisting of the principal investigator, Erin Smith, and the advising investigators, Nigel Harris, Lance Dalleck, and Gary Van Guilder. This TSM will meet every 5–6 weeks via a mix of teleconference and personal meetings. The group will discuss the current trial status as well as any decision-making for the upcoming 5–6 weeks.

#### Composition of the data monitoring committee, its role, and reporting structure {21a}

Due to the low risk, complexity, and scope of the intervention, data and safety monitoring will be performed throughout the entire trial by the TSM. However, a data and safety monitoring board (DSMB), consisting of external members with clinical and methodological expertise, will become involved if an unlikely adverse event occurs. In this case, the board will receive a report of the event and advise the TSM on the continuation and modification of the trial.

#### Adverse event and reporting and harms {22}

An adverse event is defined as a medical condition occurring between the time of consent and the last trial visit that results in the inability to participate in the intervention. A serious adverse event is defined as any untoward medical occurrence that results in an immediately life-threatening condition, severe or permanent disability, prolonged hospitalization, or significant hazard as determined by the DSMB. Any adverse event or serious adverse event between the time of participant consent and the last trial visit will be recorded on the WCU HAP Lab Incident Report Form at the time of the event. If the adverse event occurs after the time of consent but before receiving the intervention, the event will be recorded as not related to the study. If the event occurs between the first and last intervention visit, the event will be reported to the DSMB. In the case of a serious adverse event, the event will be reported to the Institutional Review Board (IRB) at Western Colorado University and Ethics Committee at Auckland University of Technology. Investigators and DSMB will determine the relatedness of an event to the study intervention. Adverse events deemed related to the study will be reported in the results of the study.

#### Frequency and plans of auditing trial conduct {23}

Trial conduct will be audited every 5–6 weeks at the TSM meeting. In the case of an adverse event, the DSMB will audit the trial conduct and advise continuation and modification.

#### Plans for communicating important protocol amendments to relevant parties {25}

Any modifications to the protocol which may impact the conduct of the study and the potential benefit of the participants or may affect participant safety, including changes of study objectives, study design, participant population, sample sizes, study procedures, or significant administrative aspects will require a formal amendment to the protocol. Such amendment will be agreed upon by the principal investigators and approved by the Ethics Committee/Institutional Review Board of WCU and AUT prior to implementation.

Administrative changes of the protocol that are minor corrections and/or clarifications that have no effect on the way the study is to be conducted will be agreed upon by the principal investigators and documented in a memorandum.

#### Dissemination plan {31a}

Approximately four papers will be submitted for journal publication from the trial results. One to two abstracts will be presented at the American College of Sports Medicine annual conference. Additionally, all participants will be informed of the trial results in a personal letter from the principal investigator.

## Discussion

Approximately 1 in 3 people in the USA suffer from MetS [7]. This statistic has increased significantly in the past decades and is projected to continue in an upward trend [7]. Similar prevalence and increasing rates have been found across the globe [101]. A plethora of evidence exists demonstrating that physical activity and exercise reduce the risk factors of MetS [4, 8–10], and therefore, exercise recommendations have been published internationally [8, 22]. Despite this evidence, only 1 in 4 adults in the world and less than 25% of adults in the USA meet these recommendations [22, 51], claiming the perceived barriers of “lack of time” and “lack of enjoyment” in the prescribed exercise recommendations [52–54]. HIFT is a time-efficient modality of exercise that has been shown to elicit enjoyment and adherence [55]; improve oxygen capacity, insulin resistance, and muscular strength [58], as well as the MetS *z*-score [59] in at-risk populations; and improve cardiorespiratory fitness [60, 61], muscular strength, and power [62], as well as waist circumference and agility [61] in healthy populations. This study aims to continue the investigation of HIFT as it seems to combat the barriers to regular exercise and improve cardiometabolic risk factors.

Researchers of this modality have used several terms to define it (e.g., HIIT-type, high-intensity circuit resistance training, multimodal high-intensity training, high-intensity interval neuromuscular training, functional high-intensity training); all terms trying to describe a modality of exercise that combines aerobic and functional resistance training performed in an interval fashion and completed in a time-efficient manner. These modalities seem to be trying to achieve a similar thing, to combat the barriers to exercise and optimize the physiological responses to achieve comprehensive health and fitness benefits. Recently, a paper was published synthesizing this initiative and modality into common terminology, high-intensity functional training (HIFT), in order to formalize a definition for better access and comparison of future studies [57]. HIFT emphasizes functional, multi-joint movements via both aerobic and muscle-strengthening exercises [57]. These exercises (e.g., squats, push-ups, pull-ups, deadlifts, weighted carry) are designed to improve the pattern of movement for activities of daily living, specific occupational tasks, and sports skills [57, 74]. Traditionally, these exercises are prescribed in sets and reps with long recovery periods and are said to have a minimal aerobic response [102]. But if performed in a high-intensity interval fashion, this modality can elicit aerobic and anaerobic adaptations as well as improve strength and power [102].

Cardiovascular fitness, muscular strength and endurance, neuromotor fitness, and cardiometabolic health are all components improved if one adheres to the published exercise recommendations (150 min/week-moderate aerobic training + 2–3 days/week-resistance training + daily flexibility training) [8, 22]. The time commitment to meet these recommendations is upwards of 250–300 min/week. For special populations, such as those with MetS, the recommendations increase the aerobic exercise to anywhere between 300 and 420 min/week [8]. If adherence to the general guidelines is poor due to the time commitment, a larger time commitment could likely increase that barrier. HIFT protocols seem to range between 20 and 40 min of exercise time, resulting in approximately 50–60 min per session if the warm-up, rest periods, and cool-down are included [57–63]. The HIFT interventions within the literature that demonstrated fitness and health improvements were performed 3–4 times per week, requiring a time commitment of 150–200 min, significantly less than the published recommendations [57–63]. However, it is unknown if health benefits are seen when HIFT is performed less than 3 times per week, therefore requiring even less time commitment. Thus, the exploration of this study.

Minimal dose, or minimal time commitment, exercise protocols are currently being explored aiming to combat the “lack of time” barrier to exercise [103, 104]. Most interventions modify modality, intensity, and/or load to therefore reduce duration and frequency, which ultimately reduces time commitment. For example, when aerobic intensity was increased to maximum, the duration of the session necessary for equivalent cardiorespiratory benefits was 67% less time than exercise at moderate intensity [105, 106]. Equivalent improvements in the markers of muscle lipid and carbohydrate oxidation were also found between the high-intensity and moderate-intensity groups [105]. The workload performed by the moderate-intensity group followed the general exercise guidelines of 150 min/week [8, 105], which are the same recommendations for blood lipid and insulin resistance improvements [107]. Although blood lipids and insulin resistance were not measured, it is possible these could be improved due to the mitochondrial remodeling seen, however with 67% less time commitment in the high-intensity group [105]. In another study investigating HIIT frequency, when high-intensity aerobic exercise (10 × 60s cycling at 83% peak power; ~ 20 min session) was performed only two times per week, significant improvements were seen in cardiorespiratory fitness and body composition [65].

Resistance training studies have also explored the minimal dose necessary for increases in strength and hypertrophy. To achieve gains, investigators have determined that resistance loads can vary (30–70% 1RM) as long as they are performed with high effort near volitional or momentary muscular failure [108]. When loads reached high amounts, the repetitions to failure were reduced, therefore reducing the time commitment [108]. These authors also determined that two of these training sessions per week was the minimum frequency to see increases in strength and hypertrophy [108]. In another dose-response study, sedentary adolescent males performed 1, 2, 3, 4, or 5 sets of a HIIT protocol (4 × 20s all-out exercise interspersed with 10-s rests), twice weekly plus one resistance training session per guidelines [8, 109]. Surprisingly, the group that performed 1 set (3 min 40s per week of HIIT) had equivalent decreases in visceral fat as the groups performing up to 5 sets (18 min 20 s per week of HIIT) [109]. Maximal oxygen uptake was improved in all groups with only a 1% greater improvement in the groups performing 4 and 5 sets [109]. These findings suggest that meaningful health benefits are gained with an extremely low dose of maximal HIIT twice weekly, plus one resistance training session [109]. All of these protocols require much less of a time commitment than the general guidelines state, yet still seem to provide beneficial metabolic and fitness adaptations.

To our knowledge, a minimal dose of exercise to see improvements in blood lipids, insulin resistance, and endothelial function has not been explored. In clinical practice, the current recommended exercise dose for blood lipid and insulin resistance is in line with the general recommendations, noting that higher volumes lead to further improvements [107]. Minimal dose hypotheses are worth testing, however, based on the demonstrated acute physiological responses, as insulin, glucose, and lipid changes have shown to last as long as 48 h after exercise [96, 110, 111]. The acute physiological responses to one session of exercise are the stimulus that leads to chronic adaptions when repeated and therefore should not be viewed in isolation [112].

Willis et al. quantified the intensity and energy expenditure of a typical HIFT workout in healthy men and women. During this 44-min exercise bout, the participant’s average HR was 80% of HR_max_ (range 69–100%), and the average energy expenditure was approximately 485 kcal (range 418–552 kcal) [63]. Although these authors did not measure any cardiometabolic markers, this has been acutely explored in other exercise interventions of the individual components of HIIT and resistance training. In healthy men performing an exhaustive cycling bout (~ 8 mins) at a high aerobic intensity of 93.5% ± 5% VO_2max_ yet lower energy expenditure (141 kcal ± 64.9 kcal) than a HIFT bout, saw significant decreases in TC and LDL-C immediate and 1-h post-exercise [113]. Also, in healthy men, when running intensity was lower (70% VO_2max_), immediate decreases in TC and LDL-C were not seen until an energy expenditure of 1300 kcal was reached, which took approximately 94 mins [114]. As the aerobic intensity and energy expenditure of a HIFT workout falls between the thresholds of these two studies, perhaps there is a sweet spot where beneficial acute blood lipid improvements can be seen. The research on acute blood lipid responses to resistance training is quite limited. One study exploring VLDL-TG kinetics the morning after evening exercise in untrained but healthy men, the authors found a 28% decrease in VLDL-TG concentration and a 30% increase in particle clearance rate after a 90 min resistance exercise bout expending ~ 400 kcal [115]. This demonstrates that resistance training has a potent acute effect on blood TG and could provide additional benefits when combined with acute lipid effects from high-intensity aerobic exercise, as is done in HIFT [8, 57].

Acute blood lipid responses seem to differ however in hypercholesterolemic (HC) men and women. Acute improvements in HDL-C and TG are commonly seen [95, 111, 116]. However, TC and LDL-C have shown an immediate decrease [95, 116], then return to baseline at 24 h [95] or rose above baseline at 24 h [116]. In another study of a 350-kcal cycling session at 80% VO_2max_, TC and LDL-C were elevated at 24 and 48 h after the session in HC men [96]. It is still unknown how hypercholesterolemic individuals may acutely respond to higher intensity intervals, as each of these studies explored steady-state exercise. “Exercise snacking” is a term to describe very short bouts of exercise (≤ 5 min) performed intermittently throughout the day and is often studied as a method to interrupt sedentary behavior [117]. The authors exploring this minimal dose approach found that 3 min of simple resistance like exercises (half-squat, calf raises, gluteal contractions, knee raises) performed every 30 min (total of 36 min/day) attenuated postprandial blood glucose, insulin, and triglyceride responses in type 2 diabetics [118].

The exercise-mediated improvements in cardiometabolic health are often not fully explained by the traditional risk factors of blood lipids, glucose, and insulin, but rather can be attributed to improvements in vascular health [45]. In untrained individuals with metabolic syndrome, one session of high-intensity aerobic training (4 × 4 min at 90–95% HR_max_ with 3 min rests between intervals) improved endothelial function from 5 to 11% immediately after, with a lasting effect for 72 h [119]. Perhaps the aerobic intensity, energy expenditure, combined modality, and time to completion of HIFT will be an ideal combination of factors to elicit acute lipid, glucose, insulin, and endothelial changes that could lead to chronic adaptations if performed frequently. The minimal dose of this repetition is unknown though. With the most commonly stated barrier to exercise as “lack of time,” minimal effective exercise dose investigations are highly warranted.

Not only is HIFT time-efficient, but HIFT is translatable to various physical settings. The equipment required is minimal, portable, and affordable compared to sophisticated cardio and resistance training machines found in most fitness facilities [57, 120]. Most HIFT protocols involve exercises with bodyweight, kettlebells, medicine balls, dumbbells, resistance bands, suspension bands, slam balls, stability balls, and other portable weighted equipment. The use of these types of “free weights” was concluded as #4 in the top 20 exercise trends of 2020 [56]. HIFT is popular [56], enjoyable [55], time-efficient [57], translatable to various settings [57], and elicits health and fitness benefits [57–63]. The results of this study will provide insight into the minimal dose of HIFT necessary for cardiometabolic health improvements in people with MetS. In turn, these findings will aid in the development of new exercise programming guidelines for MetS and related disorders.

## Trial status

Recruitment will commence in March 2022. The estimated completion date is March 2023.

## Abbreviations

MetS: Metabolic syndrome
ASCVD: Atherosclerotic cardiovascular disease
HIFT: High-intensity functional training
NHANES: National health and nutrition examination survey
HDL-C: High-density lipoprotein cholesterol
LDL-C: Low-density lipoprotein cholesterol
TG: Triglyceride
TC: Total cholesterol
VO_2max_: Maximal oxygen consumption
1RM: 1 repetition max
ApoB: Apolipoprotein B
HIIT: High-intensity interval training
HbA1c: Glycosylated hemoglobin
BP: Blood pressure
RBP: Resting blood pressure
HR: Heart rate
RHR: Resting heart rate
S_a_O_2_: Blood oxygen saturation
FBG: Fasting blood glucose
LMPP: Lipoprotein metabolism profile
INS: Fasting blood insulin
EF: Endothelial function
HOMA: Homeostatic model assessment of insulin resistance
DEXA: Dual X-ray absorptiometry
WC: Waist circumference
Ht: Height
Wt: Weight
RPE: Rate of perceived exertion
RIR: Repetitions in reserve
IFIS: International Fitness Scale
PACES: Physical Activity Questionnaire
WCU: Western Colorado University
AUT: Auckland University of Technology
HAP Lab: High Altitude Performance Lab
IRB: Institutional Review Board
GVH: Gunnison Valley Health Hospital
